# A novel three-dimensional quantitative assessment method for abnormal root morphology of the maxillary premolars in vivo on cone-beam computed tomography

**DOI:** 10.1186/s12903-022-02258-3

**Published:** 2022-06-09

**Authors:** Jian Liu, Xiao Xu, Xian-E. Wang, Peng-Cheng Jia, Meng-Qiao Pan, Li Xu

**Affiliations:** 1 Department of Periodontology, National Engineering Laboratory for Digital and Material Technology of Stomatology, Beijing Key Laboratory of Digital Stomatology, Peking University School and Hospital of Stomatology, #22 Zhongguancun South Avenue, Haidian District, Beijing, 100081 China; 2 Department of Second Clinical Division, National Engineering Laboratory for Digital and Material Technology of Stomatology, Beijing Key Laboratory of Digital Stomatology, Peking University School and Hospital of Stomatology, Beijing, China

**Keywords:** Cone-beam computed tomography, Root abnormality, Maxillary premolars, In vivo

## Abstract

**Background:**

Previous studies have described and recorded abnormal root morphology; however, most of these studies were based on two-dimensional periapical or panoramic radiographs, and only a few studies have quantified it. We aimed to combine two-dimensional periapical radiographs and three-dimensional cone-beam computed tomography (CBCT) to conduct qualitative judgments and quantitative analyses of normal and conical roots, and explore the clinical diagnostic method of normal and conical roots based on intraoral radiographs and CBCT.

**Methods:**

The conical root was identified visually on periapical radiographs as the clinical gold standard. All teeth were divided into the cone-rooted teeth (CRT) or normal-rooted teeth (NRT) groups. Furthermore, differences in root length (RL), root surface area (RSA), and root volume (RV) of conical and normal roots in the maxillary premolars on CBCT were compared. Receiver operator characteristic curves were generated, and the area under the curve (AUC) and cut-off values were calculated to evaluate the diagnostic value of RV, RSA, RV/RL, and RSA/RL.

**Results:**

The RSAs of NRT and CRT were 236.88 ± 27.93 mm^2^ and 207.98 ± 27.80 mm^2^, respectively (*P* = 0.000). The mean RV in the CRT group was lower than that in the NRT group, and the difference was statistically significant (253.40 ± 41.98 mm^3^ vs. 316.93 ± 49.89 mm^3^, *P* = 0.000). The RSA and RV of conical roots in single root premolars were 12.29% and 19.33% less than those of normal roots, respectively. The AUC values of RSA/RL and RV/RL were 0.87 and 0.89, respectively, and the best cut-off values were 19.61 for RSA/RL (if RSA/RL was < 19.61, the teeth were considered CRT) and 24.05 for RV/RL (if RV/RL was < 24.05, the teeth were considered CRT).

**Conclusions:**

CBCT has significant diagnostic value in the clinical evaluation of conical roots. RSA/RL and RV/RL were the best parameters with the largest AUC and high sensitivity and specificity.

## Background

The root of the tooth is a decisive factor for the function, stability, and long-term retention of the tooth. Abnormal root morphology is commonly observed in clinics, including curved, slender, short, and conical roots [1–3]. It is seldom considered an extremely important factor in the treatment of periodontal diseases. Periodontitis is a chronic infectious disease with plaque biofilm as the initial factor, although its occurrence, progression, clinical severity, and prognosis are affected by many factors, such as age, smoking, local factors (such as dental anatomy and restoration, calculus), and systemic diseases [4–7]. Among the local susceptibility factors of the host, abnormal root morphology is of concern, especially in Chinese patients with aggressive periodontitis, who show a root abnormality (RA) more commonly than do patients with chronic periodontitis or gingivitis [3, 8, 9]. Conical roots are a common abnormal root morphology of upper premolars. Lü et al. proved that abnormal root morphology is an adverse prognostic factor in patients with aggressive periodontitis [8]; they found that during the periodontal support therapy, patients with > 4 teeth with root abnormalities had a higher risk of tooth loss (OR = 3.52, 95% CI 1.06–11.76, *P* = 0.035) and annual SPT-TL (n ± SD: 0.16 ± 0.26 vs. 0.09 ± 0.27, *P* = 0.041). Abnormal root morphology often accelerates the progression of periodontitis, seriously affects the prognosis of periodontitis, and even leads to the failure of teeth and dentition to function properly. McGuire et al. showed that a poor crown-root ratio and poor root morphology were associated with tooth loss [10].

Previous studies have described and recorded abnormal root morphology [2, 9, 11–13]. The conical tooth has been described as having a root narrower in circumference, but may or may not have increased length compared to the overall sample average [1–3, 9]. The root is narrowed from the dental cervix to the apex, appearing triangular in mesial-distal plane view. However, most of these studies were based on two-dimensional periapical or panoramic radiographs, and only a few studies have quantified the abnormal morphology. Lind first described the short-rooted anomaly, which was observed when the crown-root ratio was greater than 1.1 [14]. However, as a common type of RA, the conical root has not yet been clearly defined.

Therefore, this study aimed to combine two-dimensional periapical radiographs and three-dimensional (3D) cone-beam computed tomography (CBCT) to conduct qualitative judgments and quantitative analyses of normal and conical single-root maxillary premolars. The qualitative judgment of the conical root on periapical radiographs was referred to as a clinical diagnostic criterion. This study compared the differences in root length (RL), root surface area (RSA), and root volume (RV) of conical and normal roots in the maxillary premolars on CBCT and attempted to explore the clinical diagnostic method and effective diagnostic parameters of normal and conical roots based on intraoral radiographs and CBCT.

## Methods

### Teeth selection

This study was approved by the Peking University Ethics Committee and Competent Authority (No. PKUSSIRB-202168154).

Periapical radiographs and CBCT results of the upper premolar were collected from the previous periodontitis case database of the oral radiology department in our hospital [Peking University School and Hospital of Stomatology] for analysis. Intraoral periapical radiographs were obtained using the bisecting-angle projection technique. Scans of premolars in vivo were obtained using a CBCT scanner (NewTom VG, QR s.r.l.) at 110 kV and 5 mA. The field of view was 12 cm × 8 cm, and the layer thickness was 0.3 mm. CBCT data of all patients were taken for therapeutic (such as prosthodontic and orthodontic) purposes and were not related to the study.

The inclusion criteria were as follows: patients (1) aged 18–40 years with systemic health data, (2) with > 20 remaining teeth, (3) with clear periapical radiographs and CBCT of the upper premolar, and (4) with single and intact roots of the upper premolar.

The exclusion criteria were as follows: patients (1) who were systemically unhealthy with tumour or metabolic disease; (2) with oral and maxillofacial acute inflammation or tumour; (3) with an orthodontics history; (4) with root resorption, filling in the root, or root fracture of the premolar; (5) with crowding or malposition in the premolar region; (6) with curved-rooted, syncretic-rooted, or short-rooted teeth; and (7) with no periodontal ligament on radiographs.

### Conical root inspection

The conical root was identified visually on periapical radiographs by three experienced periodontists. All teeth were divided into two groups, which were referred to as the cone-rooted teeth (CRT) and normal-rooted teeth (NRT) groups.

### Measurements on periapical radiographs

#### Root width parameters

**Fig. 1:**
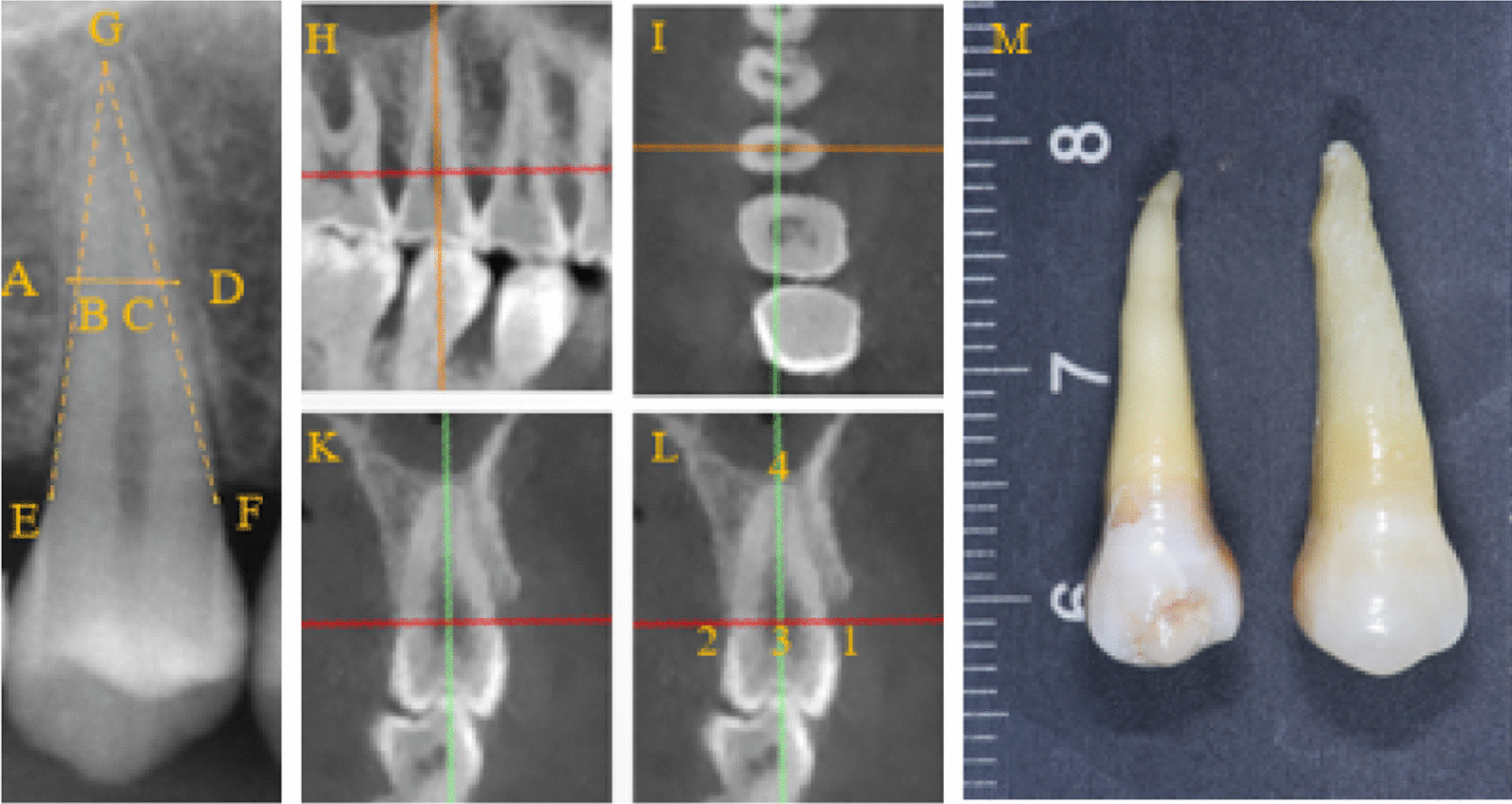
Measurements of parameters of root width and root length. GF, GE: Mesial and distal CEJ connect to apical point; B, C: Median point of GF, GE; AD: Line BC extended till the edge of the root, forming a line segment AD; H: Sagittal plane; I: Axial plane; K: Coronal plane; L:1: the lowest point of the CEJ of the buccal side; 2: the lowest point of the CEJ of the lingual side; 3: the midpoint of line 1 and 2; 4: apical root point; Line 3,4: root length; M: Conical premolar (left); Normal premolar (right)

The parameters of the upper premolars measured on periapical radiographs (Fig. 1) included the following: parameters of root width (PRW): the median points of the lines joining the mesial or distal cemento-enamel junction (CEJ) point and apex were referred to as B or C. The intersection point of the line and the mesial or distal margin of the root was defined as A or D: root width parameter = (AD − BC)/2.

### Measurements on cone-beam computed tomography

#### Root length

Mimics (Quotation Mimics 21.0, Materialise Dental) was used for the analysis. After 3D reconstruction, the images were oriented in three perpendicular planes to measure the RL on the buccal-lingual plane (Fig. 1). RL was defined as the distance between the apex and median point of the line that joins the buccal and lingual CEJ points.

#### Root surface area and root volume

**Fig. 2:**
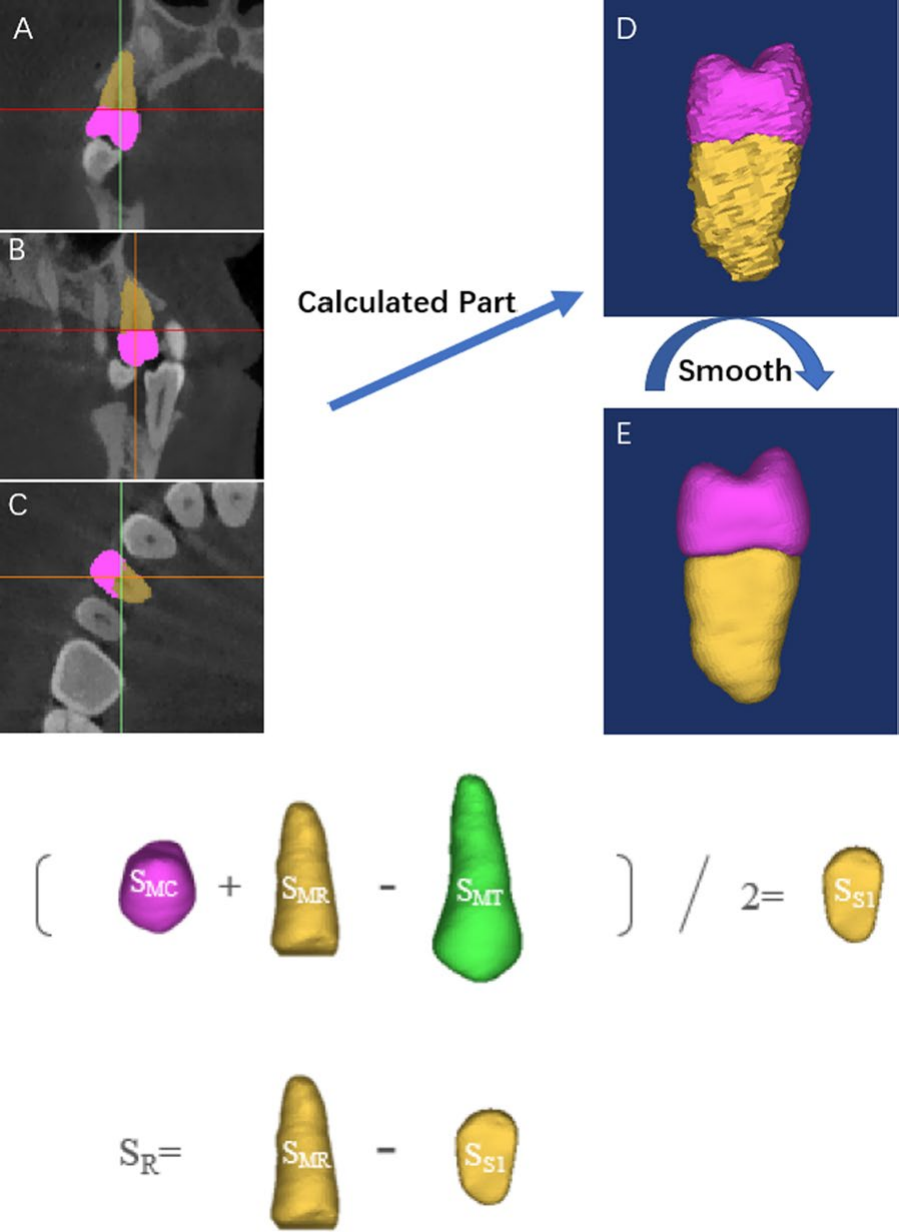
Schematic diagram of segmentation for crown, root, and tooth masks. A: Coronal plane; B: Sagittal plane; C: Axial plane; D: Initial three-dimensional image of tooth; E: Smoothened three-dimensional tooth model; SMT: Surface area of the whole tooth; SMR: Surface area of the root object; SMC: Surface area of the crown object; S_S1_: Truncation surface area; S_R_: Root surface area;

After 3D reconstruction, the margin of the premolar was determined manually from every two layers on the cross-sectional and sagittal plane images. The crown and root portions were segmented separately to the CEJ manually from each layer on the sagittal plane images. After segmentation, crown, root, and tooth masks were exported in STereoLithography format and parameters such as the RSA and RV were measured using Mimics (Fig. 2). The RSA was calculated using the formula RSA = (S_MT_ + S_MR_ − S_MC_)/2 [15]. The RV can be calculated automatically using software. RSA/RL and RV/RL were calculated manually.

### Raw colour map

All 3D original surface models from the same teeth (upper first premolar or upper second premolar) of different patients were unified in a common coordinate system and loaded in Geomagic (Geomagic Wrap 2017, 3D System). The average 3D surface models, which were the average of original models of the same teeth, were created by achieving the position of the minimum distance of the corresponding marker points through coordinate transformation under the principle of the least squares method. Furthermore, 3D differences between normal and conical root teeth were calculated to assess the morphological differences.

To help visualise the differences, a 3D, colour-coded map for the differences between the normal and conical root teeth models was generated, where the normal root teeth were displayed as smaller (negative, blue), the same (0 surface distances, green), or larger (positive, red) than the conical root teeth. The blue to red colour-coded scale was standardised, allowing a proper comparison, and pure blue and red were set at − 1.145 mm and 1.145 mm, respectively.

**Fig. 3:**
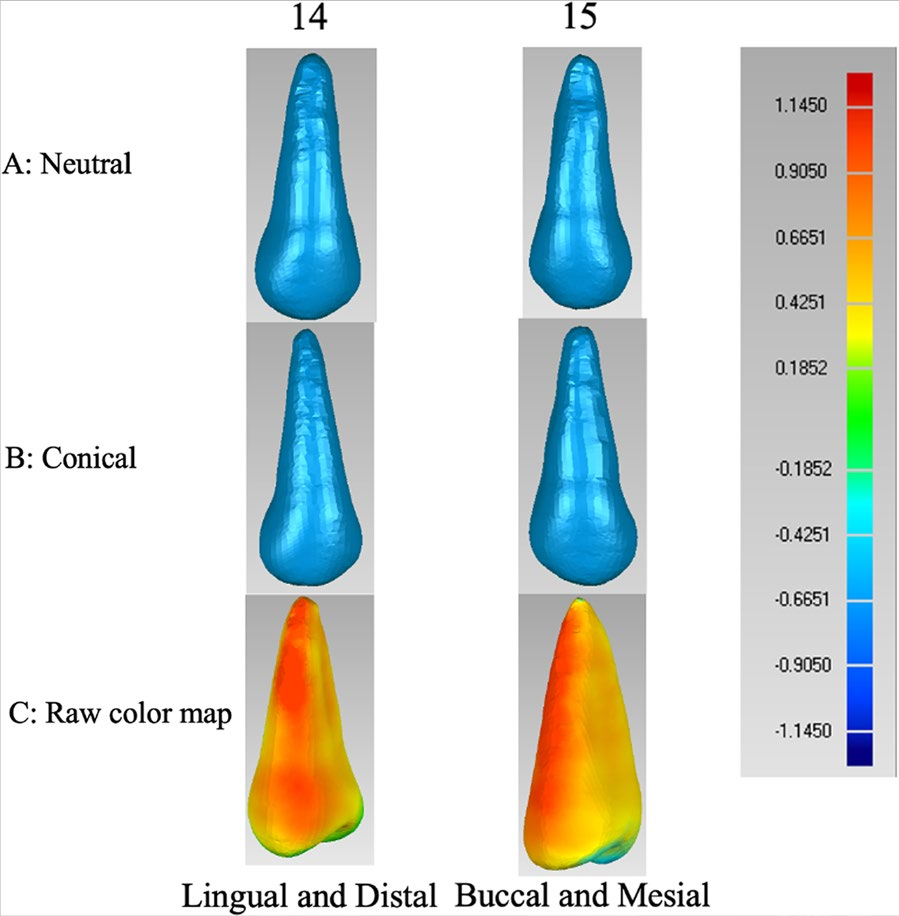
Raw colour map of the maxillary first and second premolars. **A** Average models for the neutral maxillary premolars; **B** Average models for the conical maxillary premolars; **C** Raw colour map showing the regions of statistically significant surface-to-surface differences (cone as a reference)

The raw colour map of the average models for NRT and CRT showed regions of statistically significant surface-to-surface differences (Fig. 3).

### Sample size

Based on the following formula, the sample size of this study was calculated:

$$n_{1} :n_{2} = 1:k$$

$$n_{1} = \frac{k + 1}{k}\left[ {\frac{{\left( {z_{\alpha /2} + z_{\beta } } \right)\sigma }}{\delta }} \right]^{2}$$

$$n_{2} = kn_{1}$$

Based on the results of the preliminary trial, if $k,$ α, and β values were 0.75, 0.05, and 0.20, respectively, 54 teeth and 41 teeth were required for the NRT and CRT groups, respectively.

### Statistical analyses

All statistical analyses were performed using Statistical Package for the Social Sciences Statistics version 25 software (IBM Corp., Armonk, NY, USA). Every measurement of the periapical radiograph and CBCT scan was repeated after 1 week by the same researcher, and the intra-examiner error was tested using a paired *t*-test. The intraclass correlation coefficient (ICC) was used to evaluate the magnitude of the measurement error. The mean of the two measurements was used in this study. The group *t*-test and nonparametric test were used for statistical analysis. *P* < 0.05 was considered significant for all tests.

The RA diagnosed visually on periapical radiographs was referred to as the clinical gold standard for comparison, and the diagnostic criteria remained stable in the study. Receiver operator characteristic (ROC) curves were generated, and areas under the curves (AUCs) were calculated to evaluate the diagnostic value of PRW, RV, RSA, RV/RL, and RSA/RL. After performing statistical analyses, the cut-off values were calculated for PRW, RV, RSA, RV/RL, and RSA/RL. The accuracy of the CBCT method in detecting root abnormalities was re-evaluated using the cut-off values. Subsequently, the sensitivity (Se), specificity (Sp), positive and negative predictive values, Youden index (YI), and positive and negative likelihood ratios were calculated.

## Results

In total, 44 maxillary first premolars and 51 maxillary second premolars were included in this study. The ICC values for all parameters were greater than 0.8, indicating that the measurements had excellent reliability.

There was no statistical difference for the RL between NRT and CRT (11.62 ± 1.19 mm vs. 11.65 ± 1.54 mm, *P* = 0.91). The mean PRWs for the NRT and CRT groups were 0.48 ± 0.12 mm and 0.33 ± 0.16 mm, respectively, and the difference was statistically significant. The mean RSAs for the NRT and CRT groups were 236.88 ± 27.93 mm^2^ and 207.98 ± 27.80 mm^2^, respectively (*P* = 0.000, Table 1). The mean RV in the CRT group was lower than that in the NRT group, and the difference was statistically significant (253.40 ± 41.98 mm^3^ vs. 316.93 ± 49.89 mm^3^, *P* = 0.000, Table 1). The RSA and RV of the conical roots in single root premolars were 12.29% and 19.33% less than those of normal roots.

**Table 1:** Comparison between normal-rooted and cone-rooted teeth based on RL, PRW, RSA, RV, RSA/RL, and RV/RL

	RL	PRW	RSA	RV	RSA/RL	RV/RL
NRT	11.62 ± 1.19	0.48 ± 0.12	236.88 ± 27.93	316.93 ± 49.89	20.42 ± 1.86	27.34 ± 3.87
CRT	11.65 ± 1.54	0.33 ± 0.16	207.98 ± 27.80	253.40 ± 41.98	17.88 ± 1.31	21.76 ± 2.43
*P*	0.91	0.000	0.000	0.000	0.000	0.000

Data are presented as the mean ± standard deviation

RL, root length; PRW, parameters of root width; RSA, root surface area; RV, root volume; NRT, normal root teeth; CRT, conical root teeth

**Fig. 4:**
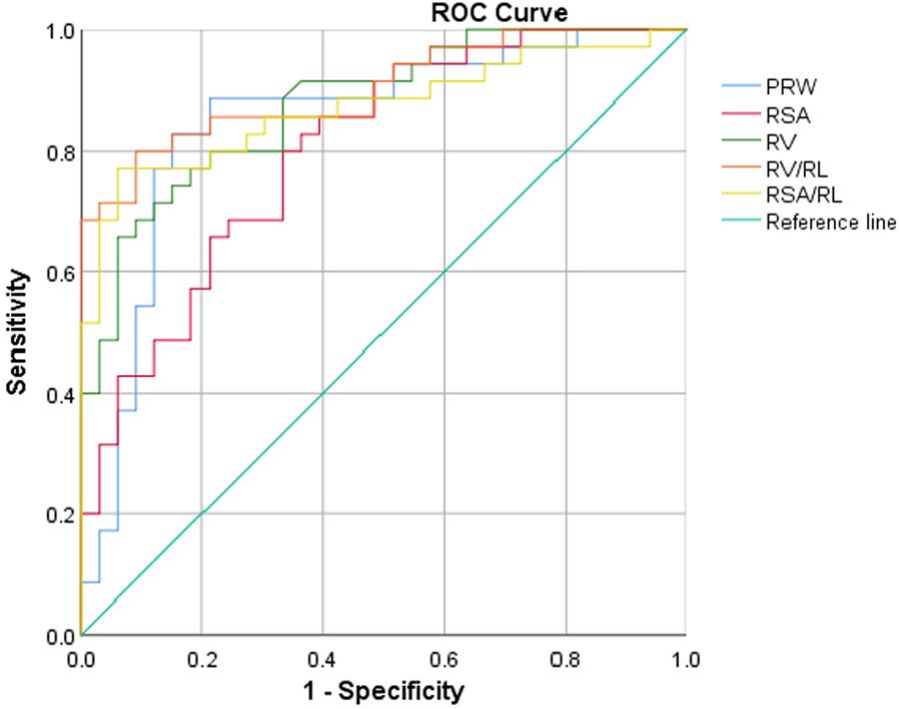
Receiver operator characteristic curves for root width, volume, surface area, and volume/length. PRW, parameters of root width; RL, root length; ROC, receiver operator characteristic curve; RSA, root surface area; RV, root volume

The ROC curves of PRW, RSA, RV, RSA/RL, and RV/RL were determined (Fig. 4). The AUC values of RSA/RL and RV/RL were 0.87 and 0.89, respectively, and the best cut-off values were 19.61 for RSA/RL (if the RSA/RL was < 19.61, the tooth was considered a conical root) and 24.05 for RV/RL (if the RV/RL was < 24.05, the tooth was considered a conical root; Table 2). The results indicated that RSA/RL and RV/RL are excellent parameters for detecting normal and conical roots. CBCT results and clinical diagnoses were reanalysed using 2 × 2 contingency tables. Based on Table 3, the accuracy of CBCT in detecting CRT was re-evaluated using the cut-off value. The Se and YI of the RSA were lower than those of other parameters, and the YI of the RV/RL was higher than that of the other parameters. RV/RL would be the most efficient parameter for the clinical detection of CRT based on the best critical point.

**Table 2:** Summary of AUCs and cut-off values for PRW, RSA, RV, RSA/RL, and RV/RL

	PRW	RSA	RV	RSA/RL	RV/RL
AUC	0.80	0.76	0.84	0.87	0.89
Cut-off	0.37	208.44	295.20	19.61	24.05
*P*	0.000	0.000	0.000	0.000	0.000

AUC, area under the curve; PRW, parameters of root width; RSA, root surface area; RV, root volume; RL, root length

**Table 3:** Accuracy of PRW, RSA, RV, RSA/RL, and RV/RL for detecting normal-rooted and cone-rooted teeth

	PRW	RSA	RV	RSA/RL	RV/RL
Se	0.73	0.51	0.83	0.95	0.88
Sp	0.85	0.89	0.72	0.72	0.81
PV+	0.79	0.78	0.69	0.72	0.78
PV−	0.81	0.71	0.85	0.95	0.90
YI	0.58	0.40	0.55	0.67	0.69
LR+	4.94	4.61	2.99	3.42	4.74
LR−	0.31	0.55	0.24	0.07	0.15

PRW, parameters of root width; RSA, root surface area; RV, root volume; RL, root length; Se, sensitivity; Sp, specificity; PV+, positive predictive value; PV−, negative predictive value; YI, Youden index; LR+, positive likelihood ratio; LR−, negative likelihood ratio

The raw colour map of the average models for NRT and CRT showed regions of statistically significant surface-to-surface differences (Fig. 3). As can be observed from the map, the red regions focused on the buccal and lingual root surfaces, and the yellow regions focused on the interproximal root surface. The results suggested that the overall dimension of the conical root was smaller than that of the normal root and not limited to the mesial-distal diameter.

## Discussion

The initial root form is a key factor associated with the prognosis of periodontitis and tooth loss16 [8, 16, 17]. As an important local factor affecting the progression of periodontitis, we should pay attention to root morphology, especially for patients with aggressive periodontitis, whose RA is more common than patients with chronic periodontitis [3, 8, 9, 18]. The conical root is a typical type of root morphological abnormality that differs from the normal root in shape and dimensions [1, 3]. However, until now, a quantitative index to diagnose conical roots from two-dimensional or three-dimensional features like short root anomaly has been lacking [14].

One aim of this study was to quantitatively evaluate the differences between conical roots and normal roots in two and three dimensions of single-root maxillary premolars. As can be observed from the results, the RSA and RV of conical roots in single root premolars were 12.29% and 19.33% less than those of normal roots without the influence of the RL, respectively, which suggests that the volume and surface area of the periodontal ligament of the conical root are smaller than those of the normal root. The geometric shape of the conical root is different to that of the normal root. Compared with the thick and long normal root, the conical root could bear a lower occlusal force because of less periodontal ligament. After periodontal destruction occurs, the normal occlusal force is significantly heavy to be borne by CRT, which may accelerate the progression of periodontitis.

In this study, the RV and RSA of the conical root were apparently lower than those of the normal root, which proved that the conical root differs with the normal root in morphology based on quantitative analysis. Ahlbrecht et al. evaluated the root morphology of maxillary incisors using CBCT and proved that 3D surface model construction for upper incisors is reproducible and 3D shape analysis using CBCT images allows a phenotypic characterisation of incisor root morphology, such as conical root, which refers to a root narrower in circumference that may or may not have increased length relative to the overall sample average [1]. This result is similar to that of this study. As can be observed from the raw colour map, the conical root was narrower in circumference than the normal root, which may have influenced the occlusal stress distribution in the periodontal ligament, the centre of rotation, and the centre of resistance. This hypothesis should be proven by finite element analysis in the future.

The second aim of this study was to find a relatively accurate diagnostic criterion to detect CR in maxillary premolars on imaging. The ROC analysis was applied to determine the best critical points to detect CR. For PRW on periapical radiographs, the AUC of PRW in detecting CR was 0.8. But in three dimensions, RSA/RL and RV/RL had greater AUCs than PRW at 0.87 and 0.89, respectively. When Se and Sp were considered to have equally important positions, the point on the ROC curve closest to the upper left corner was the point with the highest accuracy. For PRW, this point on the ROC curve was 0.37 mm, with the highest YI (0.58). For RSA/RL and RV/RL the cut-off value was 19.61 and 24.05, respectively, with the highest YI (RSA/RL, 0.67; RV/RL, 0.69).

Xu et al. showed a significant difference between conical and normal roots in terms of root width on periapical radiographs, and they quantitatively described the morphological differences between conical and normal roots in two-dimensional images for the first time [3]. Lü et al. also measured the parameters of root width of conical roots in maxillary premolars. The median (min–max) value was 0.26 (0.05–0.38) mm, so they assumed the reference value of PRW to be 0.39 mm [8], but a diagnostic efficacy analysis was not performed. There are few clinical studies related to conical roots, and they are mainly based on two-dimensional radiographs such as periapical and panoramic radiographs [2, 12, 13]. This method is subject to certain bias due to the angle, position, and exposure of the X-ray projection and has significant limitations because it cannot reconstruct the 3D structure of the root and evaluate 3D data, such as the RSA and RV. Ahlbrecht et al. made morphological comparisons but without quantitative evaluation [1].

With the wide application of high-precision CBCT in clinics, 3D reconstruction techniques based on CBCT have increasingly developed, which makes it possible to measure the 3D characterisation of teeth in vivo. A previous study focused on root resorption evaluation in patients with skeletal class III malocclusion and suggested that volume measurement based on CBCT provided a new sensitive method to detect root resorption [21]. The method has gradually become an important research method of root morphology, and the accuracy and reproducibility of the measurement of RSA and RV were high in a prior study [1, 22]. Jia et al. showed that 3D reconstruction using CBCT was accurate and reliable for measuring the RSA [15]. Wang et al. showed that the in vivo measurement of tooth volume by CBCT was as accurate as in vitro micro-computed tomography measurement [22].

Abnormal root morphology is not only related to the long-term effect of periodontal treatment, but also affects the root absorption during orthodontic treatment. Some studies show that atypical root shape, such as long, narrow, and deviated roots, increases the risk of apical root resorption during orthodontic treatment [2, 19]. All of these studies are based on periapical radiographs. However, other studies have drawn conflicting conclusions, and have hypothesised that abnormal root shape is not associated with root resorption [20].

We conducted quantitative analysis of conical roots in two and three dimensions, which is helpful for us to better judge the conical roots, especially for dentists. However, there are still some limitations in this study. This study only applied to maxillary premolars, and more clinical studies are needed to develop a CBCT method for detecting conical roots of other anterior teeth. The relationship between paralleling projection technique and conical beam CT could be explored. Also, it may take some time for doctors to learn how to use three-dimensional software.

## Conclusions

This study established a quantitative analysis method for tooth root morphology based on CBCT, and proved that this method is feasible and reproducible. We found that the surface area and volume of conical roots in single root premolars were 12.29% and 19.33% less than those of normal roots, respectively. RSA/RL and RV/RL have the best diagnostic efficiency for conical roots based on this method.

## Data Availability

The datasets used and/or analysed during the current study are available from the corresponding author on reasonable request. The datasets generated and/or analysed during the current study are not publicly available due patient imaging data is a matter of personal privacy.

## Abbreviations

3D: Three-dimensional
AUC: Area under the curve
CBCT: Cone-beam computed tomography
CEJ: Cemento-enamel junction
CRT: Cone-rooted teeth
ICC: Intraclass correlation coefficient
NRT: Normal-rooted teeth
PRW: Parameters of root width
RL: Root length
ROC: Receiver operator characteristic curve
RSA: Root surface area
RV: Root volume
Se: Sensitivity
Sp: Specificity
YI: Youden index
